# Supplementary material for: Abundant small RNAs in the reproductive tissues and eggs of the honey bee, Apis mellifera
Source: BMC Genomics. 2022 Apr 4;23:257. doi: 10.1186/s12864-022-08478-9 (PMC8978429; doi:10.1186/s12864-022-08478-9)
Supplement: Supplementary file 1 — Additional file 1. [file 12864_2022_8478_MOESM1_ESM.pdf]

Supplemental Data File

Supplemental Table legends.

Supplemental Table S1. Novel miRNAs and their expression pattern across tissues

Supplemental Table S2. Pairwise comparison of tRF expression across tissues

Supplemental Table S3. The global list of 199 piRNA clusters obtained after merging overlapping and proximal (within 1kB) piRNA clusters that were identified across each of the five tissue groups. Features included in this table include the coordinates, length, what tissues the cluster was identified in (indicatd by X), and strandedness. Strandedness was assigned if >50% of unique/collapsed reads mapped to one strand, otherwise the cluster was assigned as dual/bidirectional.

Supplemental Table S4. An ordered list (by fold-change) of top 10 genes that have the most antisense mapping piRNA reads, for each pairwise comparison of tissues.

Supplemental Table S5. Gene ontology information obtained for miRNA and piRNA predicted gene targets for each tissue, obtained from ShinyGO v 0.61.

Supplemental Figure 1

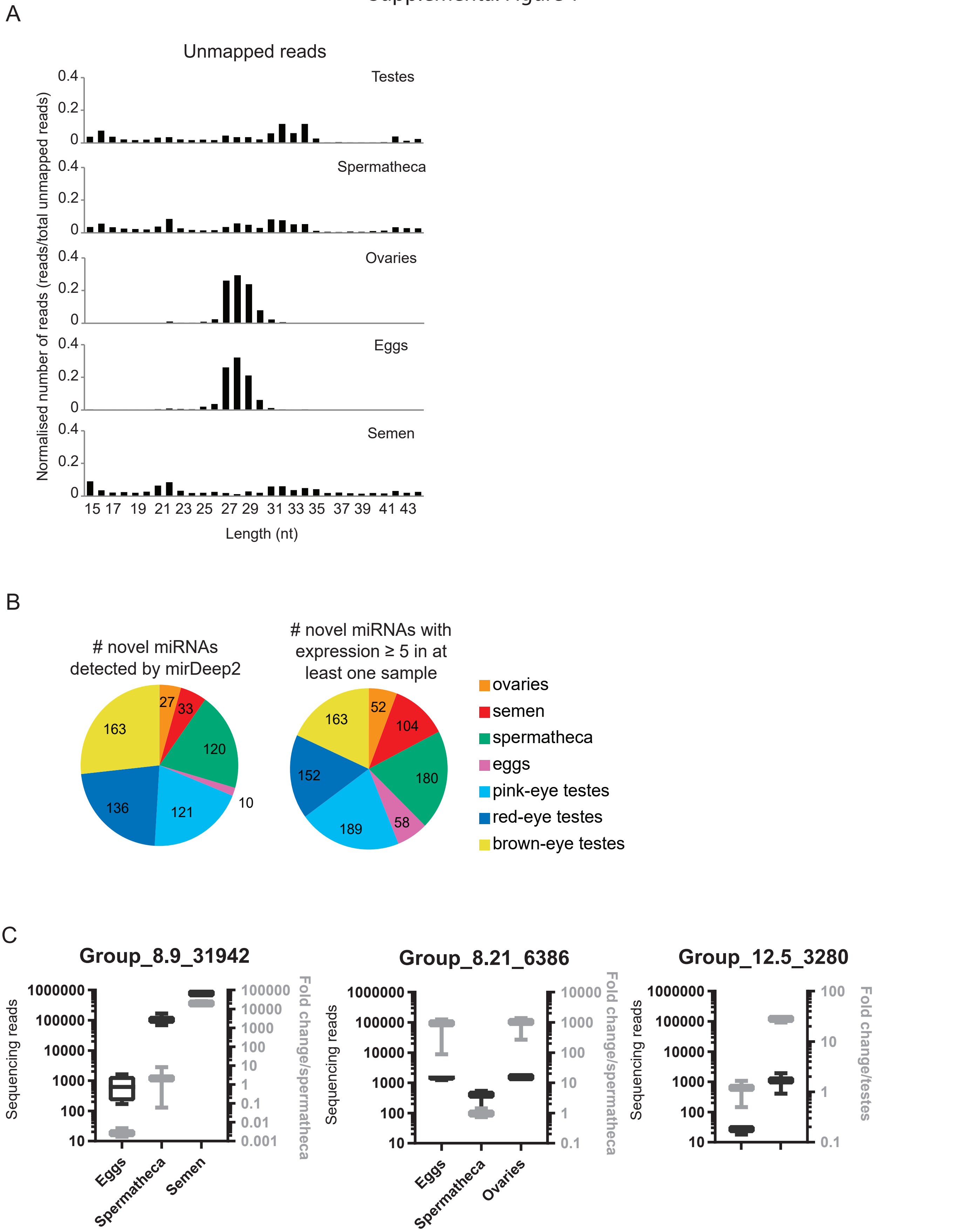

Supplemental Figure 1. A. Size distribution of total small RNAs between 13 and 43 nt, contained in the small RNA libraries, that did not map to the Amel 4.5 honey bee genome. **B**. Number of novel miRNAs detected by miRDeep2 in each tissue (left). Right panel shows the total number of novel miRNAs with expression greater than 5 in each tissue. **C**. Stemloop qPCR validation (grey- measured as fold change) of three novel miRNAs which exhibit differential tissue expression via small RNA-seq (black- measured as read count differences).

Supplemental Figure 2

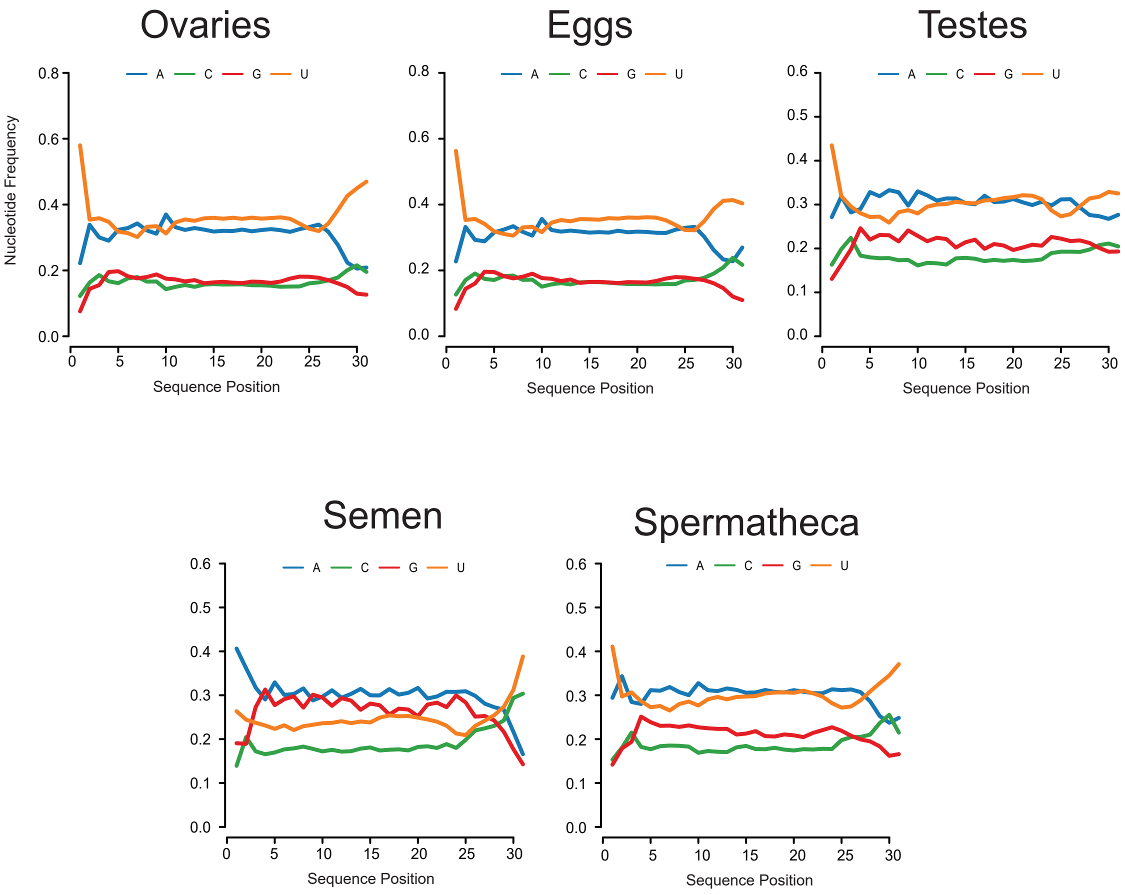

Supplemental Figure 2. Nucleotide frequency plots for each tissue using total putative piRNAs (26-31 nt length reads).

Supplemental Figure 3

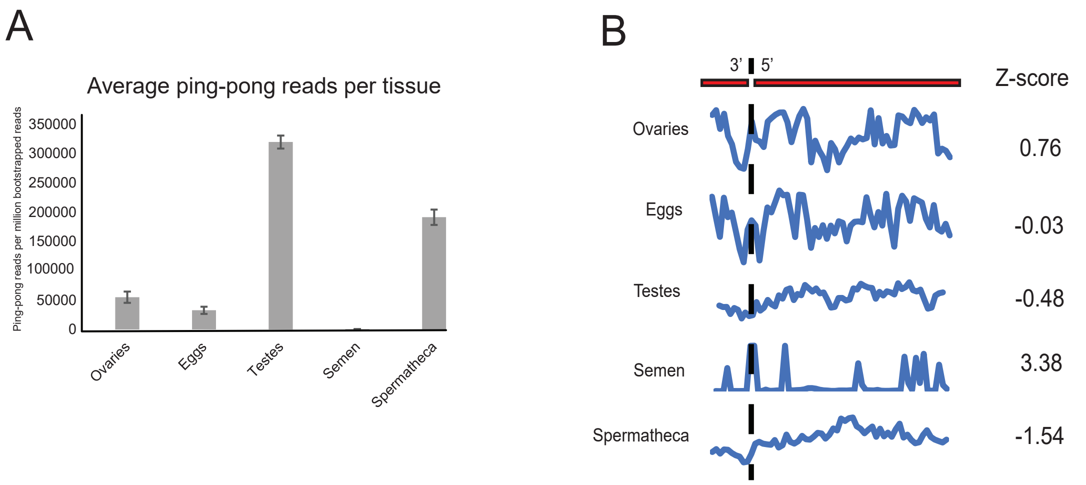

Supplemental Figure 3. A. The average number of ping-pong reads (reads that have a 10nt 5′ overlap with another mapped read) after bootstrapping using 100 pseudo-replicate datasets. B. Analysis of 3′-5′ distances of mapped reads, a peak at a distance of 1bp indicates the presence of piRNA phasing. Z scores for phasing were calculated using the method of Han et al (Han et al. 2015).

Supplemental Figure 4

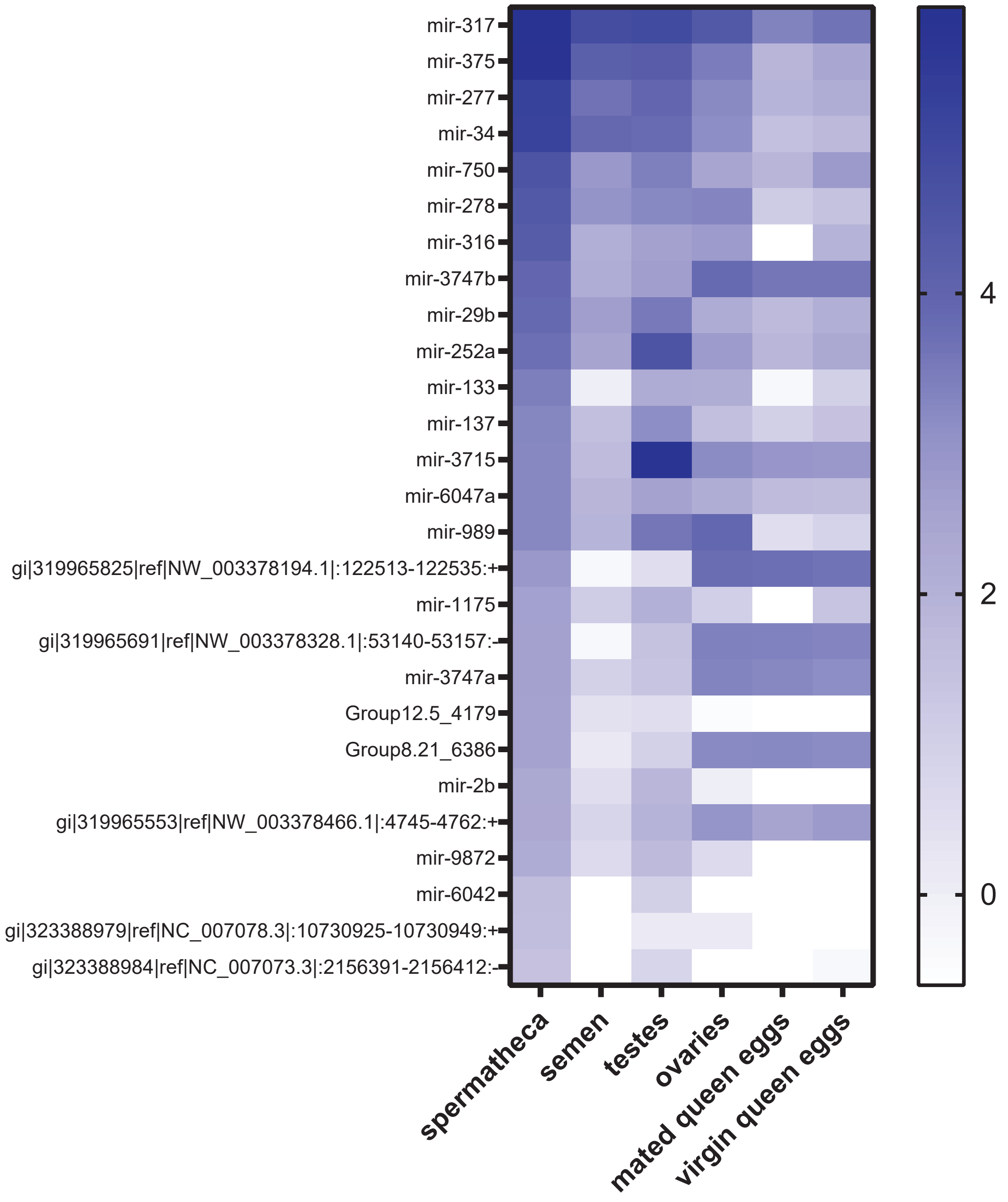

Supplemental Figure 4. Heatmap showing log fold change of miRNAs between different tissues.
